# The prevalence and correlates of alcohol use and alcohol use disorders: a population based study in Colombo, Sri Lanka

**DOI:** 10.1186/s12888-015-0549-z

**Published:** 2015-07-14

**Authors:** Helena M.S. Zavos, Sisira Siribaddana, Harriet A. Ball, Michael T. Lynskey, Athula Sumathipala, Frühling V. Rijsdijk, Matthew Hotopf

**Affiliations:** 1 Social Genetic and Developmental Psychiatry Centre, Institute of Psychiatry, Psychology & Neuroscience, Kings College, DeCrespigny Park, Denmark Hill, London, SE5 8AF UK; 2 Sri Lanka Twin Registry, Institute of Research and Development, Battaramulla, 10120 Sri Lanka; 3 Department of Medicine, Faculty of Medicine & Allied Sciences, Rajarata University of Sri Lanka Anuradhapura, Mihintale, Sri Lanka; 4 Addictions Department, Institute of Psychiatry, King’s College London, London, SE5 8BB UK; 5 Health Services and Population Research, Institute of Psychiatry, Kings College, University of London, London, SE5 8AF UK; 6 Research Institute for Primary Care & Health Sciences, Keele University, Keele, UK; 7 Department of Psychological Medicine, Institute of Psychiatry, Kings College, London, SE5 9RJ UK

**Keywords:** Alcohol use, Alcohol use disorders, Sri Lanka, Comorbidity

## Abstract

**Background:**

Alcohol use is increasing in non-Western countries. However, the effects of this increase on the prevalence of alcohol use disorders (AUD) remains unknown, particularly in South Asia. This study aimed to estimate the prevalence of alcohol use and AUD in the Colombo District, Sri Lanka. Environmental risk factors and psychiatric correlates were also examined.

**Methods:**

The Composite International Diagnostic Interview was used to assess alcohol use and psychiatric disorders in a population based sample of 6014 twins and singletons in the Colombo region of Sri Lanka.

**Results:**

Lifetime alcohol use on 12 or more occasions was estimated at 63.1 % (95 % CI: 61.3-64.9) in men and 3.7 % (95 % CI: 3.0-4.3) in women. Prevalence of lifetime alcohol abuse and alcohol dependence in men was 6.2 % (95 % CI: 5.3-7.1) and 4.0 % (95 % CI: 3.3-4.7) respectively. Lower standard of living was independently associated with alcohol use and dependence but not abuse. Significant associations between lifetime AUD and other psychiatric disorders were observed.

**Conclusions:**

Lower prevalence of alcohol use and AUD was observed compared to Western countries. Prevalence of alcohol use and AUD were higher than previous reports. Socio-demographic and environmental risk factors appear to be similar across cultures as were associations between AUD and other psychiatric disorders.

## Background

 The use and abuse of alcohol is a global health problem, with developed countries showing near ubiquitous alcohol use. Such use has an enormous public health impact [1]. Patterns of consumption and use disorders are less well understood in low and middle income countries. However, as these become increasingly urbanized and affluent, thus increasing access as well as relaxing previous social checks to alcohol consumption, it might be anticipated that alcohol consumption and use disorders will increase. Sri Lanka is a middle-income country in South Asia with lower, but increasing, rates of alcohol use compared to Western countries [2, 3]. The prevalence of alcohol use disorders (AUD) is less clear as few studies have employed structured psychiatric interviews to estimate rates of AUD and their associated problems in South Asia, and Sri Lanka specifically. It is, therefore, unclear whether cultural differences in attitudes to drinking provide a protective effect against the development of AUD amongst alcohol users. Such research has important health, economic and policy implications.

More than 60 % of men and women in Western populations have consumed at least 12 alcoholic drinks in the previous 12-months [4]. AUD are also relatively common in Western countries. For example, the 12-month prevalence of alcohol abuse and alcohol dependence in the USA has been reported as 4.7 and 3.8 % respectively [5]. Lifetime prevalence’s of these disorders were estimated at 17.8 and 12.5 % respectively [5]. Rates of alcohol use are generally lower in non-Western countries including Sri Lanka, the setting of the current study. The WHO global status report on alcohol and health [6] which used data from the WHO global survey on Alcohol & Health (2012) in addition to other surveys conducted in the respective countries estimated the prevalence of lifetime ever alcohol use in Sri Lanka at 43.1 % of men and 19.5 % of women. However, frequency of past year abstinence (person not having drunk in previous 12 months) were as high as 72.8 % in men and 90.1 % of women. The 12-month prevalence of AUD has been estimated at 5.6 % in men and 0.6 % in women [3]. Previous research suggests the low rates of alcohol use are largely explained by cultural influences [7].

Men are more likely to engage in alcohol use and are at a much greater risk of developing AUD than women throughout the world [3, 8]. While socio-economic status (SES) has consistently been associated with alcohol use in Western countries, comparatively less research has been conducted in South Asian countries and findings have been mixed. For example, some studies have suggested an association between lower income and increased alcohol use [2, 9] whereas others have shown alcohol use to be highest in upper income families [10]. Education, however, seems to be a more consistent predictor of increased alcohol use in South Asia with a number of studies showing an association between lower education levels and alcohol use [9, 11].

There is considerable evidence that AUD are frequently co-morbid with other mental disorders in developed countries [5, 12]. In developing countries, population-based studies have also shown evidence for an association between high levels of alcohol use, poor mental health (in particular internalizing problems), and suicide [13]. Similar findings have been reported in Sri-Lanka. For example, an autopsy study of suicide showed that 49 % of males who committed suicide were alcohol dependent [14]. However, to date, no study has examined the relationship between AUD and nicotine dependence and internalizing disorders in Sri-Lanka.

Given the paucity of findings surrounding AUD in South Asia, and Sri-Lanka specifically, the current study sought to describe the epidemiology of AUD in a representative sample of the Colombo district in Sri Lanka. We explored the symptom profile of AUD and investigated environmental risk factors for use and AUD. Associations between AUD and other mental health disorders were also assessed.

## Methods

### Ethics statement

The study received approvals from the Institute of Psychiatry, King’s College London Research Ethics Committee; the Ethical Review Committee, University of Sri Jayewardanepura; and the World Health Organisation’s Research Ethics Committee. In the conduct of the study, all participants were encouraged to discuss participation with family members. The consent procedure was the same for all participants including those under 18 years old and was approved by the relevant ethics committees. Participants were included only if they provided written informed consent. Data was anonymized before receipt of data and before analysis.

### Participants

Data from the Colombo Twin And Singleton Study (CoTASS) sample were used [15, 16]. The CoTASS sample is a population-based twin study with a comparable singleton sample. Data collection took place in the Colombo District of Sri Lanka, an area with a population of approximately 2.2 million, which includes the island’s capital and urban to semi-urban areas.

Twins were identified by adding a question to the annual census which asked whether the householder knew of any twins. All 13 secretariat divisions of the Colombo district were surveyed. Using this method, 19,302 twins were identified. The return rate was lowest in the most densely populated areas (Colombo (49.6 %) and Thimbirigasyaye (32.6 %)) and highest in the semi-urban areas to the East of the capital (Padukka (82.8 %) and Hanwella (88.0 %). Of these, 4,387 twins were randomly selected to take part in the CoTASS project, a total of 4,024 (91.7 %) agreed to participate. A parallel singleton population was randomly selected from the same local area from which the twins were recruited. Of those contacted (*N* = 2,311), 2,019 (87.4 %) agreed to participate. Whilst the inclusion of a twin sample is unusual in a prevalence study, because of the systematic population ascertainment of twins and exceptionally high participation rate, it is possible to use the sample to estimate population prevalence, with the assumption that twins are no different from non twins.

The twin and singleton samples were different in age (mean ages: 34 years and 43 years for twin and singleton sample respectively) but similar in sex distribution (48.2 and 45.6 % male in twin and singleton sample respectively). Within the twin sample, 90 % of participants stated their ethnicity as Sinhalese, 5.8 % Moors and 3.7 % Tamil. This was similar in the singleton sample with 96.8 % of participants stating ethinicity as Sinhalese, 1.0 % Tamil, 2.3 % Muslim and >1.0 % Burger.

Interviews were conducted by high-school-educated research workers [see 15 for more information]. Interviews took place between 2006 and 2007 when Sri Lanka had been experiencing violent civil war for over 20 years. However, much of the conflict centered in areas to the North and East of the island, some distance from the location of the current study. Nonetheless, a small minority (2.6 %) of the participants reported directly participating in the conflict as combatants. Participants were included if they consented, were over 16 years of age and spoke sufficient Sinhala to understand the interview.

### Measures

#### Alcohol use, AUD and other psychiatric disorders

Psychiatric phenotypes including alcohol abuse, alcohol dependence, nicotine dependence, depression, anxiety and PTSD were assessed using the Composite International Diagnostic Interview CIDI [17]. This is a structured diagnostic interview designed for use by lay interviewers. The questionnaire was adapted by a total of 13 bilingual twins (contacted from the registry) and other Sri Lankans fluent in English and Sinhala. Each measure was adapted at least twice independently. The translations were then reviewed in group meetings consisting of 7 professionals and checked by a bilingual scholar in Sinhala. Translations were not direct, but instead aimed to find forms of words in Sinhalese that best described the concepts of interest [see 15 for more information].

Alcohol use was assessed by asking participants whether they have ever had 12 alcoholic drinks at anytime in their life. Pictorial reference cards of typical Sri Lanka drinks were provided to guide participants on what constitutes one drink (e.g. 50 ml Arrack, Whisky or Kasippu). The cards included pictures of drinks which are brewed at home, e.g. toddy (or palm wine), which is the fermented sap of the coconut palm. Alcohol dependence is defined as a maladaptive pattern of substance use which has led to significant impairment or distress as manifested by three or more of the following symptoms occurring within the same 12 month period: 1) tolerance; 2) withdrawal; 3) loss of control 4) desire to quit; 5) preoccupation 6) give up on other activities and 7) used despite problems. Alcohol abuse is defined as a maladaptive pattern of substance use which has led to significant distress as manifested by 1 or more of the following symptoms which occur within a 12-month period: 1) failure to fulfill obligations; 2) recurrent use in hazardous situations; 3) recurrent alcohol related legal problems and 4) continued use despite persistent or recurrent social or personal problems. Individuals meeting the criteria for alcohol abuse were not classified as such if they also met the criteria for alcohol dependence. These individuals (*N* = 80) were excluded from analyses involving alcohol abuse.

### Standard of living and environmental risk

Socio-demographic characteristics and current living environment were assessed using items from the Sri Lanka census. Items spanned a wide range of household characteristics rather than concentrating on the extremes. Items included questions about: housing tenure and type; overcrowding; quality of structural materials; toilet and water facilities; lighting and fuel type; household commodities; access to means of transport; a subjective report of financial situation and experiencing hunger due to poverty in the last three months. A composite of standard of living variable was created by summing across these items; the alpha reliability was 0.8.

### Statistical analysis

The combination of the twin and singleton data was constructed in SPSS [18]. Analyses were performed in STATA [19] and as our sample included individuals from the same families, statistical analyses were conservatively corrected for the non-independence of observations using the *‘robust’ cluster* command as is standard in analyses of this type (see [20, 21] for more information).

Due to the low frequency of drinking behaviors in females (only 1 female was categorized as having an AUD), associations between alcohol use phenotypes, environmental and other psychiatric variables were only examined in male participants. Statistical analysis was carried out in three stages. First, the 12-month and lifetime prevalence of alcohol use, abuse and dependence was calculated in our sample. Lifetime prevalence was then stratified by socio-demographic characteristics for example, age and marital status. Adjusted odds ratios for all socio-demographic variables were then calculated. Second, the recency and onset of both alcohol abuse and dependence as well as the symptom profile for each disorder were examined. Finally, associations between lifetime AUD and other psychiatric disorders were examined.

## Results

### Prevalence and symptom profile

The 12-month prevalence of alcohol use was 22.7 % (95 % CI: 21.6-23.7) in the current sample (*N* = 6012). Men had a higher 12-month prevalence of alcohol use than women (Male: 48.0 % 95 % CI: 46.3-50.0; Female: 1.0 % 95 % CI: 0.7-1.4). Lifetime use of 12 or more drinks of alcohol was reported by 63.1 % (95 % CI: 61.3 - 64.9) of men and 3.7 % (95 % CI: 3.0 - 4.3) of women. Of men reporting alcohol use, the majority were relatively infrequent users (43.8 % reported drinking less than once a month) with 33.2 % reporting weekly use of alcohol. Mean age of onset for alcohol use was 20.8 years.

AUD were examined in males only due to the low prevalence of females reporting alcohol use and AUD. The 12-month prevalence of alcohol abuse was 3.1 % (95 % CI: 2.4-3.7). The prevalence of lifetime alcohol abuse was 6.4 % (95 % CI: 5.5 - 7.3) in men (10.1 % of individuals reporting alcohol use). Alcohol dependence was experienced by 4.0 % (95 % CI: 3.3-4.7) of men in the whole sample (6.4 % of men reporting alcohol use). The 12-month prevalence of alcohol dependence was 1.8 % (95 % CI: 1.3-2.3). The mean age of onset was 26.5 years for alcohol abuse and 29.6 years for alcohol dependence.

Among participants reporting alcohol abuse, the median number of symptoms endorsed was 1. The most common behavior was recurrent use of alcohol in hazardous situations with over 40 % of individuals meeting the criteria for alcohol abuse endorsing this symptom (see Table 1). For participants reporting alcohol dependence, the median number of symptoms endorsed for dependence was 4 (a total of 3 symptoms need to be endorsed in order to meet the criteria for alcohol dependence). The highest endorsements were for tolerance, loss of control and desire to quit.

**Table 1:** Endorsement of DSM-IV criteria for alcohol dependence and abuse in males

		Endorsement in males (%)	Endorsement in males meeting case definition (%)
Alcohol abuse		*N* = 2727	*N* = 170
	DSM-IV criterion		
	Failure to fulfill obligation	4.3	37.7
	Recurrent use in hazardous situations	3.7	42.9
	Recurrent alcohol related legal problems	1.1	12.9
	Continued use despite social problems	3.6	31.2
Alcohol dependence		*N* = 2726	*N* = 110
	DSM-IV criterion		
	Tolerance	11.6	88.2
	Withdrawal	3.8	48.2
	Loss of control	9.0	88.2
	Desire to quit	22.9	87.3
	Preoccupation	3.9	47.3
	Give up on other activities	2.7	38.2
	Used despite problems	4.4	58.2

Note. Alcohol abuse includes only individuals who did not also meet the criteria for alcohol dependence

### Socio-demographic factors

Socio-demographic characteristics of individuals reporting alcohol use and those who meet the criteria for alcohol abuse and alcohol dependence are given in Table 2. Age was associated with alcohol use and both abuse and dependence. Marital status and years of schooling were associated with both alcohol use and dependence but not alcohol abuse. Twin/non-twin status was also associated with alcohol dependence, but not alcohol use or abuse, with lower prevalence associated with being a member of a twin pair. Significantly more Sinhalese participants reported alcohol use, however, there was no association between ethnicity either alcohol abuse or dependence. Urbanicity was not associated with any of the alcohol use phenotypes.

**Table 2:** The prevalence of lifetime alcohol use, dependence and abuse in males among different socio-demographic groups

Characteristic	Alcohol users	Alcohol abuse	Alcohol dependence	Alcohol users compared to non-users	Alcohol abuse compared to non- abusers	Alcohol dependent compared to non-dependent
	n/N (%)	n/N (%)	n/N (%)	Adjusted OR^a^	Adjusted OR^a^	Adjusted OR^a^
*Whole sample (95 % CIs)*	1729/2730 (63.1)	170/2730 (6.2)	110/2729 (4.0)			
	(61.5-65.1)	(5.3-7.1)	(3.3-4.8)			
*Twin Status*						
Twin *(reference)*	1063/1819 (58.4)	116/1819 (6.4)	59/1818 (3.3)	-	-	-
Non-twin	669/911 (73.7)	54/911 (5.9)	51/911 (5.6)**	1.4 (1.1-1.7)**	.96 (0.7-1.4)	1.5 (.96-2.3)
*Age (quartiles)*						
1^st^ (16–24 yrs) *(reference)*	267/730 (36.9)	25/730 (3.4)	11/730 (1.5)	-	-	-
2^nd^ (25–34 yrs)	470/672 (69.9)**	54/672 (8.0)**	29/672 (4.3)**	2.6 (1.8-3.7)**	3.1 (1.7-5.9)**	3.3 (1.4-7.6)**
3^rd^ (35–46 yrs)	456/605 (75.4)**	48/605 (7.9)**	30/604 (5.0)**	2.2 (1.3-3.7)	4.4 (1.8-10.9)**	4.1 (1.3-12.8)*
4^th^ (47–89 yrs)	534/721 (74.1)**	42/721 (5.8)*	40/721 (5.6)**	1.3 (0.6-3.1)	5.2 (1.3-20.1)*	4.9 (1.0-24.4)
*Ethnicity*						
Sinhala *(reference)*	1635/2519 (64.9)	156/2519 (6.2)	106/2518 (4.2)	-	-	-
Non-Sinhala	94/211 (44.6)**	14/211 (6.6)	4/211 (1.9)	0.4 (0.3-0.6)**	1.2 (0.6-2.3)	0.5 (0.2-1.3)
*Marital status*						
Married *(reference)*	1204/1609 (74.8)	111/1609 (6.9)	75/1608 (4.7)	-	-	-
Never married	485/1074 (45.2)**	56/1074 (5.2)	29/1074 (2.7)*	0.6 (0.4-0.8)**	1.1 (0.7-1.7)	1.2 (.6-2.3)
Previously married	38/45 (84.4)	2/45 (4.4)	6/45 (13.3)*	1.6 (0.7-3.7)	.74 (0.2-3.2)	2.7 (1.0-7.1)*
*Years of schooling*						
Up to 10 *(reference)*	696/976 (71.3)	69/976 (7.1)	58/975 (6.0)	-	-	-
11-12	611/987 (61.9)**	61/987 (6.2)	32/987 (3.2)**	0.82 (0.7-1.0)	0.88 (0.6-1.3)	0.6 (0.4-1.0)*
13+	405/738 (54.9)**	38/738 (5.2)	19/738 (2.6)**	0.63 (0.5-0.8)	0.79 (0.5-1.2)	0.50 (0.3-0.9)*
*Urbanicity*						
Semi-urban *(reference)*	1017/1645 (61.8)	105/1645 (6.4)	61/1644 (3.7)	-	-	-
Urban	711/1084 (65.6)	65/1084 (6.2)	49/1084 (4.5)	1.4 (1.2-1.7)**	.93 (0.7-1.3)	1.27 (0.8-1.9)

Note. Asterisks show unadjusted association: *p < 0.05, **p < 0.01. Previously married = widowed/separated/divorced

^a^Adjusted for all variables in this table, plus age as a continuous variable. Alcohol abuse includes only individuals who did not also meet the criteria for alcohol dependence (*N* = 80). Due to power constraints, analyses based on ethnicity have been collapsed into Sinhala and non-Sinhala categories

When looking at the adjusted odds ratios, twin status, age, years of schooling remain significant predictors of alcohol use. The effects of urbanicity on alcohol use became significant once other covariates are taken into account. Adjusted odds ratios show age to be the only characteristic independently associated with both alcohol abuse and dependence. However, results also showed that years of schooling and marital status were still associated with alcohol dependence once other socio-demographic factors were controlled for. Twin status was not associated with alcohol dependence or abuse once other covariates were taken into account.

### Standard of living

The composite of standard of living variable was significantly associated with alcohol use and alcohol dependence (see Table 3). Specifically, lower standard of living was associated with higher prevalence of alcohol use and dependence. Standard of living was not associated with alcohol abuse. The population attributable fraction which indexes the proportional reduction of population alcohol use/AUD if exposure to low standard of living was eliminated, ranged between 0.1 for alcohol abuse and 0.4 for alcohol dependence.

**Table 3:** The relationship between standard of living and alcohol use, dependence and abuse in males

Standard of living variable	*N*	%	Unadjusted OR	Adjusted OR^a^	PAF
Alcohol Use					
*SoL (standardized)*			1.2 (1.1-1.3)	1.2 (1.1-1.3)	
*SoL quintiles*					
Top 3 quintiles	1022	61.1			
Bottom 2 quintiles	705	66.8	1.3 (1.1-1.5)	1.2 (1.0-1.4)	0.2
Alcohol Abuse					
*SoL (standardized)*			1.2 (1.0-1.3)	1.1 (1.0-1.3)	
*SoL quintiles*					
Top 3 quintiles	99	5.9			
Bottom 2 quintiles	70	6.6	1.2 (0.8-1.5)	1.0 (0.8-1.5)	0.1
Alcohol Dependence					
*SoL (standardized)*		-	1.5 (1.3-1.7)	1.4 (1.2-1.7)	
*SoL quintiles*					
Top 3 quintiles	41	2.5			
Bottom 2 quintiles	69	6.5	2.8 (1.9-4.2)	2.6 (1.7-4.0)	0.4

Note.

Alcohol abuse includes only individuals who did not also meet the criteria for alcohol dependence (*N* = 80). *PAF* population attributable fraction, *SoL* higher scores indicate lower standard of living

^a^adjusted for age, education, urbanicity, marital status and ethnicity

### Associations between AUDs and other psychiatric disorders

The associations between lifetime alcohol abuse and dependence and other psychiatric disorders, controlling for socio-demographic characteristics and psychiatric co-morbidity are given in Table 4. As the prevalence of anxiety disorder subtypes was low, with the exception of generalized anxiety disorders, they were combined. Lifetime alcohol abuse was associated with nicotine dependence, depression and generalized anxiety but not anxiety overall or PTSD. Lifetime alcohol dependence was associated with greater prevalence of nicotine dependence, depression, anxiety and PTSD. Odds ratios were reduced when controlling for other co-morbidity. All associations with alcohol abuse and alcohol dependence, with the exception of anxiety, remained significant.

**Table 4:** Adjusted odds ratios of lifetime AUD and other psychiatric disorders.

	Adjusted Odds Ratios (95 % confidence intervals)					
	Sociodemographic characteristics^a^			Sociodemographic Characteristics and other psychiatric disorders^b^		
	Alcohol abuse	Alcohol dependence	Any AUD	Alcohol abuse	Alcohol dependence	Any AUD
Nicotine dependence	2.7 (1.6-4.6)**	11.2 (7.0-18.0)**	7.1 (4.6-10.5)**	3.7 (2.1-6.4)**	10.8 (6.4-18.0)**	6.1 (4.0-9.1)**
Depression	2.3 (1.3-3.9)**	6.3 (3.7-10.7)**	4.3 (2.9-6.5)**	2.7 (1.5-4.8)**	4.9 (2.6-9.1)	3.5 (2.2-5.4)**
Any Anxiety	1.6 (.87-2.9)	3.4 (2.0-5.8)**	2.5 (1.6-3.8)**	1.3 (0.7-2.5)	1.2 (0.5-2.7)	1.3 (0.7-2.2)
Generalized anxiety	3.0 (1.3-6.7)**	6.7 (3.3-13.4)**	5.6 (3.1-10.2)**	3.2 (1.6-6.6)*	3.3 (1.3-8.5)**	3.2 (1.3-7.6)**
PTSD	1.5 (0.5-4.2)	9.7 (4.6-20.7)**	5.3 (2.7-10.4)**	1.6 (0.5-5.4)	4.6 (1.8-12.2)**	2.9 (1.3-6.3)**

**p < 0.01, *p < 0.05

^a^adjusted for age, education, urbanicity, marital status and ethnicity

^b^adjusted for age, education, urbanicity, marital status and ethnicity and other psychiatric disorders

## Discussion

The 12-month and lifetime prevalence of alcohol use was found to be significantly lower in the current Sri Lankan sample than reports in Western countries. This was especially notable in females where the lifetime prevalence of alcohol use was less than 4 %. The pronounced gender difference in alcohol consumption could be due to a number of reasons [7, 13, 22]. First, the use of alcohol is generally seen as a more masculine habit in Sri Lanka and other Asian countries. Second, women may show higher adherence to the religious guidelines, such as strong opposition to the use of any addictive substance in Buddhism, the major religion in Sri Lanka [22]. However, it is also possible that gender differences can be explained in part by a tendency for women to feel less comfortable reporting alcohol use.

Whilst the prevalence of alcohol use was lower than in Western countries, it was higher than previous estimates In Sri Lanka [3], despite our use of a more stringent measure. The upward trend in alcohol consumption may be due to a number of socio-cultural factors including urbanization, westernization, as well as availability and affordability of alcohol [10]. There is also some evidence to suggest that alcohol distributors are targeting youth in an effort to increase sales in this region [23, 24]. However, our finding may also be due to the relatively young age of the sample and the fact that the sample is from Colombo rather than being a national sample.

Due to the low prevalence of alcohol use in females, AUD were only examined in men. Consistent with previous research in South Asia and Sri Lanka, we found lower prevalences of both alcohol abuse and dependence than reports in US and Europe. For example, the 12 month prevalence of alcohol abuse and alcohol dependence was 3.1 and 1.8 % respectively, compared to 6.9 and 5.4 % in US males [5]. This difference is not just explained by a smaller proportion of the Sri Lankan population consuming alcohol: the prevalence of AUD amongst alcohol users was also substantially lower than reports in Western populations (lifetime prevalence of abuse and dependence in male users was 10.1 and 6.4 % respectively in the current study compared to 24.6 and 17.4 % in US sample [5]). This low rate of AUD even amongst alcohol users suggests that culture may provide a protective effect from developing AUD in Sri Lanka. However, it should be noted that the prevalence of AUD are higher than previously reported. For example, a previous study conducted in the 1980s reported a prevalence of 1.3 % of alcoholism [25].

### Criterion endorsement

In line with previous research [26], we found that the majority of individuals with alcohol abuse tended to endorse only one symptom dimension. The most common criterion endorsed was hazardous use (42.9 %) followed by role interference and social problems. The most commonly endorsed symptoms of alcohol dependence in the current sample were tolerance, loss of control and desire to quit. This is similar to a multi-site study [27] as well as results from a nationally representative sample in the US [28]. This suggests that the symptom profile of alcohol abuse and dependence in Sri Lanka is similar to that in the West.

### Socio-economic correlates and environmental associations

As the prevalence of 12 month AUD was very low we were only able to examine socio-demographic risk factors in relation to lifetime prevalence of alcohol abuse and dependence. A number of socio-demographic factors were associated with lifetime alcohol use including ethnicity, marital status and years of schooling. Socio-demographic risk factors were similar for alcohol dependence and abuse, and overall the pattern was very similar to research done in Western countries suggesting similar risk factors are associated with maladaptive alcohol consumption in these different cultures.

A significant association between low standard of living and alcohol use was observed in the current study. This is in line with previous research in Sri Lanka, and other south Asian countries, which showed greater alcohol use in lower income groups [2, 9]. Other studies have, however, found alcohol use to be highest in upper income families [10]. This suggests that more research in this area is necessary to fully determine the relationship between SES and alcohol use in Sri Lanka.

When looking at AUD, standard of living was associated with alcohol dependence but not alcohol abuse (individuals at the lower end of the distribution at greater risk of alcohol dependence). Previous research looking at the motivations for alcohol use in young Sri Lankan males has found that tension reduction was the most prominent motivation for drinking (drinking for personal enjoyment or due to social pressure did not significantly predict drinking frequency) [10]. This raises the possibility that men drinking as a way to cope with their social and economic problems. The finding is also concerning as research has shown that although low income earners spend less on alcohol than high income earners, the proportion of income spent is higher and may compromise their ability to meet basic needs [29]. Due to the cross-sectional nature of the data, we are unable to determine whether the direction of effects goes from low standard of living to increased alcohol use or vice versa.

### Association between alcohol use disorders and other psychiatric disorders

There is a growing body of work investigating the associations between AUDs and other psychiatric disorders. The results of the current study are similar to co-morbidity studies in the West and indicate that individuals with an alcohol use disorder are significantly more likely to suffer from a range of other psychiatric problems. When controlling for socio-demographic factors, AUDs were associated with increased prevalence of nicotine dependence, depression, general anxiety and PTSD.

The overlap between alcohol use problems and other psychiatric problems could indicate similar aetiological factors in the development of such problems. For example, previous research has implicated similar socio-demographic and environmental influences for depression as for alcohol use problems [30]. Shared genetic mechanisms may also be important with some twin studies demonstrating genetic overlap between alcohol use problems and nicotine use [31] as well as internalizing problems [32, 33]. Other research has, however, implicated a causal model whereby problems with alcohol have led to depression, or alternatively a self-medication model in which depression leads to increased the risk of AUD [34].

### Strengths and Limitations

We used a well validated structured interview to ascertain alcohol use and AUD, had a population sample and very high participation rates. There are, however, some limitations. First, whilst the sample was large, the prevalence of some of the disorders under study was low and some of the estimates are therefore imprecise. Further, the data are cross sectional making it impossible to determine the direction of causation between SES, other psychiatric disorders and AUD. Our sample consisted of both twins and singletons and whilst they were matched in terms of locality there were some differences between them on for relevant variables including alcohol use which may affect the generalisability of the results. Finally, the results focused on the Colombo district and may not, therefore, be generalisable to other parts of Sri Lanka. Although our results suggested no influence of urbanicity on AUD – rural populations have lower consumption.

## Conclusions

The prevalence of alcohol use and AUD are lower in Sri Lanka than in Western countries but appear to be increasing. In spite of the cultural differences in attitudes towards drinking, the symptom profile of AUD and environmental correlates in Sri Lanka are very similar those reported in Western populations. Alcohol consumption is placing an increasing burden on developing countries. As AUD are greater in individuals with a lower standard of living it may exacerbate the problems caused by poverty. The significant correlations evident between AUD and other psychiatric problems including mood disorders further emphasizes the importance of tackling alcohol misuse.
